# Design and In Vitro Evaluation of a Cytotoxic Conjugate Based on the Anti-HER2 Affibody Fused to the Fc Fragment of IgG1

**DOI:** 10.3390/ijms18081688

**Published:** 2017-08-03

**Authors:** Alicja M. Sochaj-Gregorczyk, Patryk Ludzia, Emilia Kozdrowska, Piotr Jakimowicz, Aleksandra Sokolowska-Wedzina, Jacek Otlewski

**Affiliations:** 1 Department of Protein Engineering, Faculty of Biotechnology, University of Wroclaw, 50-383 Wroclaw, Poland; alicja.sochaj-gregorczyk@uj.edu.pl (A.M.S.-G.); patryk.ludzia@icloud.com (P.L.); emilia.kozdrowska@gmail.com (E.K.); aleksandra.sokolowska-wedzina@uwr.edu.pl (A.S.-W.); 2 Department of Protein Biotechnology, Faculty of Biotechnology, University of Wroclaw, 50-383 Wroclaw, Poland; piotr.jakimowicz@uwr.edu.pl

**Keywords:** affibody, HER2, Fc fragment of IgG1, monomethyl auristatin E (MMAE)

## Abstract

In our previous work we demonstrated that a small protein called affibody can be used for a cytotoxic conjugate development. The anti-HER2 affibody was armed with one moiety of a highly potent auristatin E and specifically killed HER2-positive cancer cells with a nanomolar IC_50_. The aim of this study was to improve the anti-HER2 affibody conjugate by increasing its size and the number of conjugated auristatin molecules. The affibody was fused to the Fc fragment of IgG1 resulting in a dimeric construct with the molecular weight of 68 kDa, referred to as Z_HER2:2891_-Fc, ensuring its prolonged half-life in the blood. Due to the presence of four interchain cysteines, the fusion protein could carry four drug molecules. Notably, the in vitro tests of the improved anti-HER2 conjugate revealed that it exhibits the IC_50_ of 130 pM for the HER2-positive SK-BR-3 cells and 98 nM for the HER2-negative MDA-MB-231 cells. High efficacy and specificity of the auristatin conjugate based on Z_HER2:2891_-Fc indicate that this construct is suitable for further in vivo evaluation.

## 1. Introduction

Safe and efficient delivery of cytotoxic payloads to solid tumors remains one of the main challenges for advanced cancer therapies. An antibody-drug conjugate (ADC) consists of a monoclonal antibody that recognizes tumor-associated antigens covalently linked to a highly cytotoxic agent. This composition confers a limited systematic exposure to the drug minimizing adverse effects of the therapy [1,2]. Since the 1980s, many ADCs have undergone clinical trials and currently, more than 60 are being tested [3,4,5,6]. Nevertheless, only two ADCs, brentuximab vedotin (Adcetris, Seattle Genetics) against Hodgin’s lymphoma and ado-trastuzumab emtansine (Kadcyla, Genentech) against metastatic breast cancer, were approved for marketing by the Food and Drug Administration (FDA) and are being used to treat patients.

Conventional ADCs employ monoclonal antibodies as targeting molecules. Recently, the feasibility of other molecules to deliver cytotoxic payloads or radionuclides to cancer cells has been addressed in an increasing number of studies [7,8]. Among the antibody alternatives are antibody fragments such as scFvs (28 kDa) and Fabs (55 kDa) [9,10,11,12], nanobodies (12–15 kDa) [13,14,15,16], affimers (12–14 kDa) [17], signaling molecules [18] and affibodies (6–7 kDa) [19,20,21,22,23,24]. Affibodies are engineered proteins based on a three-helix bundle Z domain that was derived from the IgG-binding B domain of protein A from *Staphylococcus aureus* [25]. Randomization of 13 surface-exposed residues present in the first two alpha-helices followed by phage display allowed for the generation of affibodies that recognize and bind various molecular targets with high affinity [26,27,28]. One of the most studied affibodies is a variant selected against HER2 receptor (Human Epidermal Growth Factor Receptor 2) [29,30,31]. This receptor is overexpressed in 20–25% of metastatic breast cancer cases [32]. The main advantages of affibodies over antibodies in terms of targeted therapy and molecular imaging include their small size that ensures better penetration of solid tumors, high stability, lack of disulphide bridges and low-cost manufacturing in a bacterial expression system.

**Figure 1:**
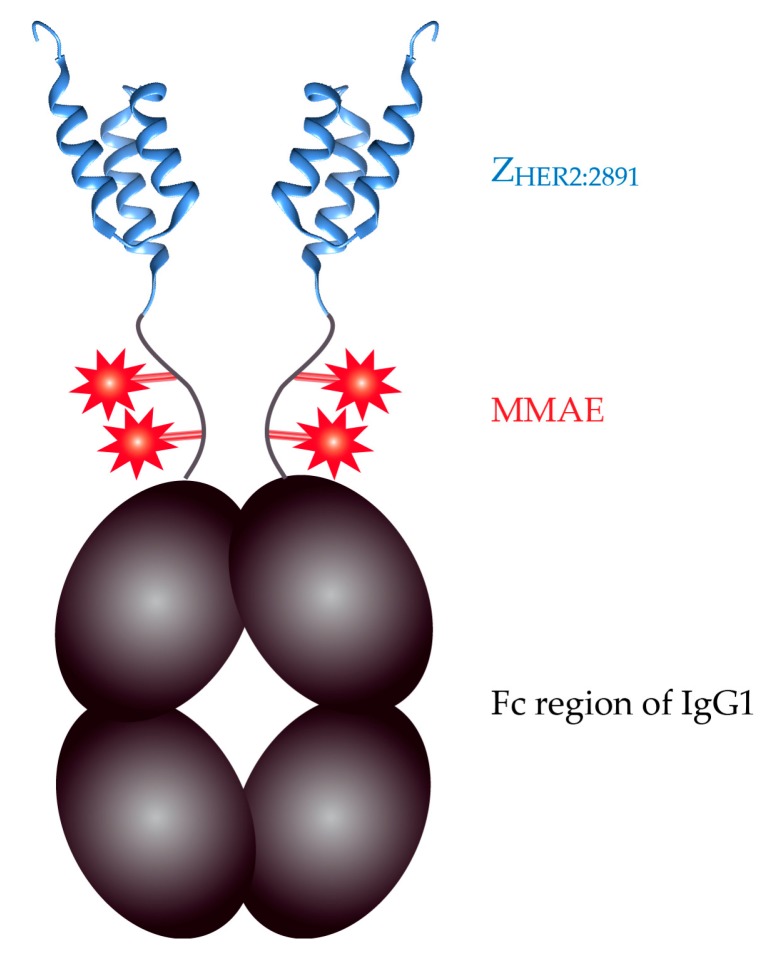
Scheme of the Z_HER2:2891_-Fc-MMAE homodimer.

In our previous study, we demonstrated that the anti-HER2 affibody Z_HER2:2891,_ originally designed by Feldwisch et al. (2010) efficiently delivers a highly cytotoxic antimitotic agent, auristatin E, to HER2-positive cancer cells [19]. However, the small size of this conjugate (8647 Da) would result in its fast renal clearance as the molecules smaller than 25–40 kDa are rapidly removed from circulation via kidney filtration [22,33]. In order to expand the size of our conjugate and increase its half-life in blood, we decided to fuse the Z_HER2891_ affibody to the Fc fragment of IgG1 [34,35,36]. The resulting Z_HER2:2891_-Fc fusion homodimerizes via Fc has the molecular mass of 68 kDa, which should contribute to its retention in the circulation. Additionally, the Fc fragment ensures that the conjugate will be recycled from epithelial cells back to blood vessels via interaction with FcRn [37,38]. Moreover, the presence of two interchain disulphide bonds in the Fc part allowed us to conjugate four auristatin molecules to our dimeric construct (Figure 1) whereas the previous Z_HER2:2891_-DCS-MMAE conjugate was loaded only with a single auristatin molecule. The cytotoxicity of our improved construct referred to as Z_HER2:2891_-Fc-MMAE, was evaluated on breast cancer cell lines.

## 2. Results

### 2.1. Expression and Purification of the Z_HER2:2891_-Fc from Mammalian Cells

**Figure 2:**
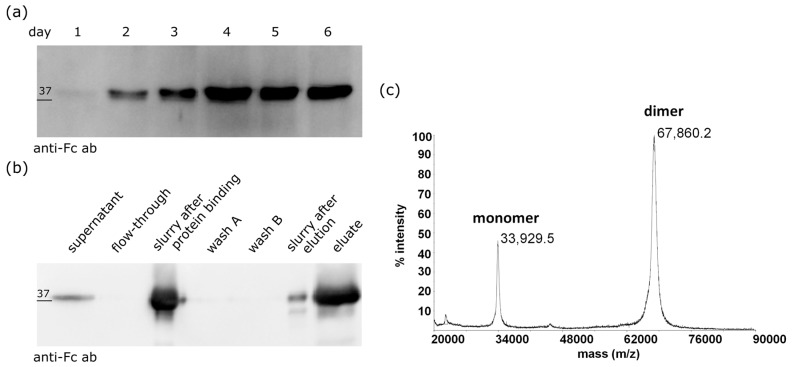
Expression and purification of Z_HER2:2891_-Fc. (**a**) The levels of Z_HER2:2891_-Fc in the CHO-S cells culture medium; (**b**) Western blot analysis of the purification of Z_HER2:2891_-Fc; (**c**) The mass spectrum of the purified Z_HER2:2891_-Fc.

The conjugate developed here is based on the molecule named Z_HER2:2891_, which exhibits increased thermal stability and hydrophilicity along with lower liver uptake in animals when compared to the parental Z_HER2:342_ affibody [30,39]. To expand the size of our construct, we fused the Z_HER2:2891_ molecule to the Fc fragment of IgG1 (Figure 1). A similar fusion was previously proposed by Rönnmark et al. (2002) who fused the Tag DNA polymerase specific affibody to the Fc fragment and purified this construct from *E. coli* cells [40]. However, the Z_HER2:2891_-Fc fusion protein was expressed in Chinese Hamster Ovary CHO-S cells to ensure its proper folding and glycosylation [41,42]. The highest levels of Z_HER2:2891_-Fc in the culture medium were observed since day 4 following the transfection with pLEV113-Z_HER2:2891_-Fc (Figure 2a). Therefore, Z_HER2:2891_-Fc was purified on day 5 or day 6 using a single-step affinity chromatography on protein A-Sepharose. The purification process was analyzed by Western blotting with the anti-Fc antibody conjugated with HRP (Figure 2b). The Z_HER2:2891_-Fc fusion migrated as a 36-kDa band in SDS-PAGE in the presence of the reducing agent, β-mercaptoethanol (5%), in Laemmli sample buffer. Additionally, the identity and purity of the product were confirmed by mass spectrometry (MS) (Figure 2c). The detected *m*/*z* peaks corresponded to a covalent dimer (67,860.2 Da) and monomer (33,929.5 Da) of the Z_HER2:2891_-Fc after cleavage of a 19 amino acid-long secretion signal peptide (2273.7 Da). We obtained 1.5 mg of the Z_HER2:2891_-Fc protein from 1 liter of CHO-S culture.

### 2.2. Specificity of the Z_HER2:2891_-Fc Fusion Protein

**Figure 3:**
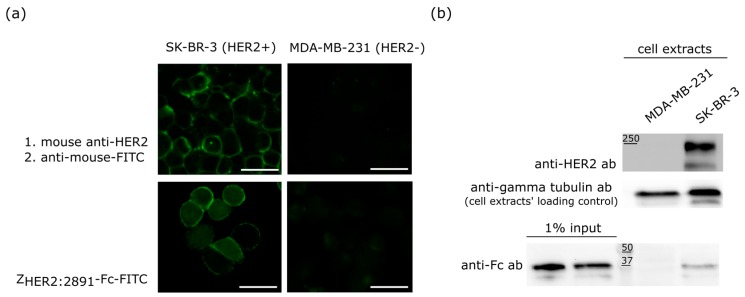
Specificity of the Z_HER2:2891_-Fc binding. (**a**) Fluorescent microscopy images of the HER2-positive SK-BR-3 and MDA-MB-231 HER2-negative cells. The anti-HER2 antibody was detected with a FITC-labeled secondary antibody whereas the affibody was FITC labelled, scale bar = 100 µm; (**b**) Z_HER2:2891_-Fc binding to the SK-BR-3 and MDA-MB-231 cells analyzed by Western blotting. The upper panel shows HER2 levels in SK-BR-3 and MDA-MB-231cells, the middle panel severs as a loading control for the experiment, the bottom panel demonstrates that Z_HER2:2891_-Fc is specifically enriched at the surface of the SK-BR-3 cells.

Our previous study clearly demonstrated that Z_HER2:2891_ selectively recognized HER2 receptor present on the surface of cancer cell lines that overexpress this receptor [19]. Since the proposed fusion contains the same targeting molecule, we verified the specificity of Z_HER2:2891_-Fc towards two out of four cell lines that were further used to assess cytotoxicity of monomethylauristatin E (MMAE) coupled to Z_HER2:2891_-Fc. We chose the SK-BR-3 and MDA-MD-231 breast cancer cells as they express very high and low, physiological levels of HER2 receptor, respectively [19,43,44]. The Z_HER2:2891_-Fc was labeled with FITC and used for the immunostaining experiment. The fluorescent signal was detected on the surface of SK-BR-3 cells when Z_HER2:2891_-Fc–FITC or anti-HER2 antibody were used to recognize HER2 receptor (Figure 3a). In the case of the HER2-negative MDA-MB-231 cells, only a weak background signal was observed. We also analyzed Z_HER2:2891_-Fc binding to SK-BR-3 and MDA-MB-231 cells by Western blotting with anti-Fc antibody-HRP. The band corresponding to Z_HER2:2891_-Fc was present only in the case of the HER2-positive SK-BR-3 cells (Figure 3b). Taken together, these biochemical and microscopic results are consistent with the analysis of Z_HER2:2891_ binding to different cell lines carried out in our previous study [19] and confirm that the fusion protein specifically recognizes HER2 receptor.

### 2.3. The Z_HER2:2891_-Fc-MMAE Conjugate

**Figure 4:**
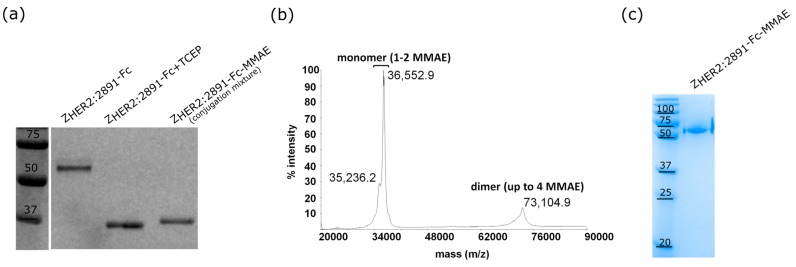
Conjugation of MMAE to Z_HER2:2891_-Fc. (**a**) The Z_HER2:2891_-Fc species before and after conjugation separated on 12% SDS-PAGE gel and subjected to Coomassie staining. The samples were heated (96 °C, 10 min) in non-reducing Laemmli buffer prior the electrophoretic separation; (**b**) The mass spectrum of the conjugation product; (**c**) The Z_HER2:2891_-Fc-MMAE conjugate migration on a native 12% polyacrylamide gel under non-reducing conditions stained with Coomassie [45].

Reduction of the two interchain disulphide bonds between the Fc fragments of the fusion protein resulted in four –SH groups which reacted with the maleimide group present in the drug molecule with a high efficiency (~90%) (Figure 4a). In the MS spectrum recorded for the conjugate, two main *m*/*z* peaks were present. The major *m*/*z* peak (36,552.9 Da) corresponds to a monomeric Z_HER2:2891_-Fc loaded with two MMAE molecules whereas the second *m*/*z* peak (73,104.9 Da) represents a dimeric Z_HER2:2891_-Fc carrying up to four auristatin E (MW 1316.7 Da). Our analysis also revealed a trace of the Z_HER2:2891_-Fc monomer conjugated with one MMAE molecule (*m*/*z* = 35,236.2) (Figure 4b). The strong signal detected for the monomeric conjugate loaded with one or two MMAE can be attributed to the breakdown of the non-covalent conjugate dimer since the disulphide bridges were replaced with MMAE molecules. Such noncovalent dimer is prone to decomposition during sample preparation and exposure to a laser beam in the MS experiment. To confirm that the conjugate dimerizes, we subjected Z_HER2:2891_-Fc-MMAE to a polyacrylamide gel electrophoresis with a low concentration of SDS (0.0375%) in the running buffer under non-reducing conditions [45]. The conjugate migrated as a band of a molecular mass between 50 and 75 kDa, which demonstrates that the dimeric format of Z_HER2:2891_-Fc is preserved in its auristatin conjugate (Figure 4c).

### 2.4. In Vitro Efficacy of the Z_HER2:2891_-Fc-MMAE

**Figure 5:**
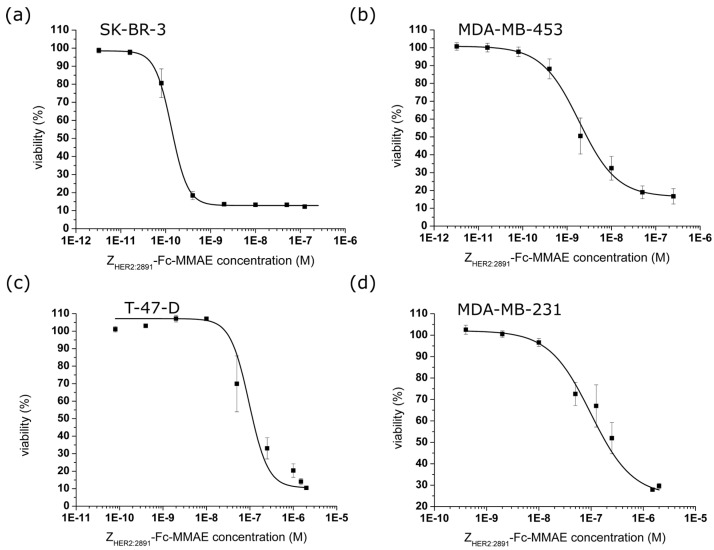
Cytotoxicity of the Z_HER2:2891_-Fc-MMAE conjugate. The conjugate was tested on four breast cancer cell lines: (**a**) SK-BR-3 (HER2+); (**b**) MDA-MB-453 (HER2+); (**c**) T-47-D (HER2-) and (**d**) MDA-MB-231 (HER2-). Error bars represent standard error of the mean (SEM).

Our previous conjugate based on a single Z_HER2:2891_ with the C-terminal extension, referred to as drug conjugation sequence (DCS), could carry only a single MMAE moiety. The IC_50_ of this construct determined for SK-BR-3 cells, which overexpress high levels of HER2 receptor was ~5 nM [19]. Since the Z_HER2:2891_-Fc-MMAE contained two affibody molecules and was armed with four drug molecules, we had expected to observe a significant decrease in the IC_50_ value. The cytotoxicity assays were carried out exactly the same way as for Z_HER2:2891_-MMAE and free MMAE in our previous study [19]. In order to more precisely compare HER2 expression levels between used breast cancer cell lines: MDA-MB-231, T-47-D, MDA-MB-453 and SK-BR-3, we carried out a densitometric quantification on the anti-HER2 Western blots published previously [19]. According to our analysis, HER2 levels in MDA-MB-453 were three to five times higher than in T-47-D cells and about six to eight times lower than in SK-BR-3 cells. We did not detect the band corresponding to HER2 in MDA-MD-231, which confirms that this is a strong HER2-negative line. For clarity, we decided to divide the used cell lines for HER2-positive (SK-BR-3 and MDA-MB-453) and for HER2-negative cell lines (MDA-MB-231 and T-47-D) which is in accordance with literature [43,44]. These four cell lines were subjected to different concentrations of Z_HER2:2891_-Fc-MMAE and the cell viability was determined with the Alamar Blue assay after 96 h of the continuous exposure to the conjugate. The IC_50_ values corresponded to the HER2 level of tested cells. Consequently, SK-BR-3 cells exhibited the lowest IC_50_ value of 134 pM, the IC_50_ obtained for MDA-MB-453 cells was 1.9 nM whereas IC_50_ values for T-47-D and MDA-MB-231 were 45.7 nM and 98.2 nM, respectively (Figure 5 and Table 1). Notably, these results show that we significantly improved in vitro efficacy of our new anti-HER2 affibody-based conjugate in comparison to the previous Z_HER2:2891_-DCS-MMAE construct (Table 1).

### 2.5. Stability of the Z_HER2:2891_-Fc-MMAE Conjugate in Serum

**Figure 6:**
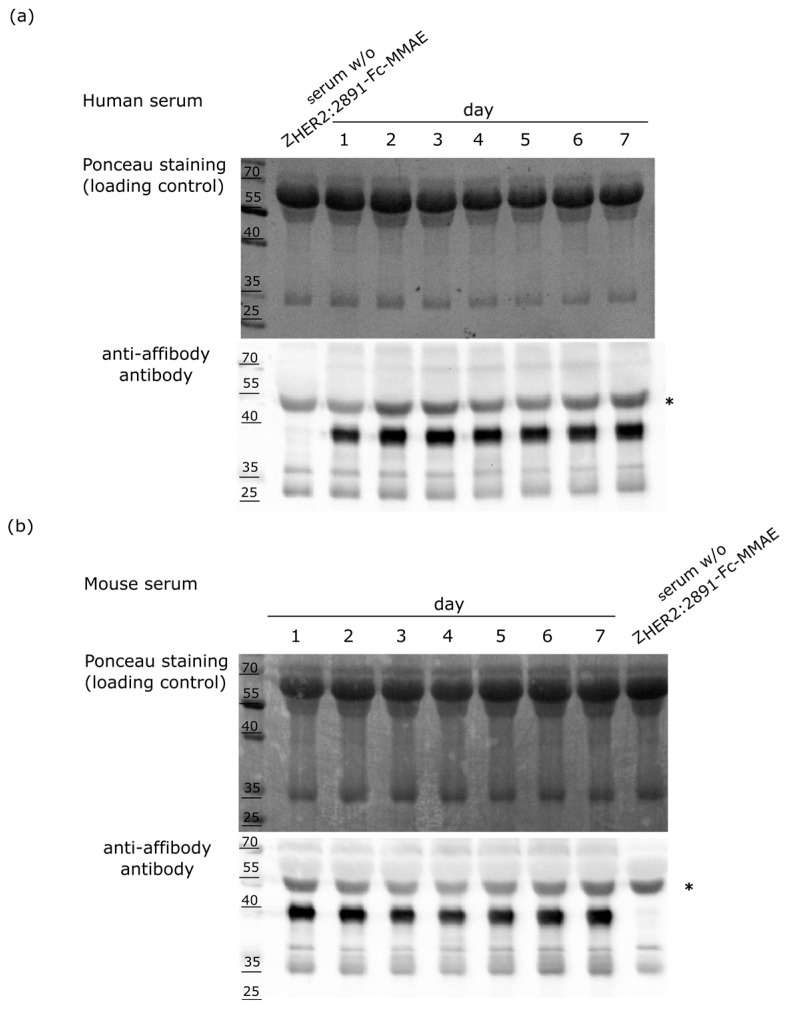
The stability of Z_HER2:2891_-Fc-MMAE in (**a**) human and (**b**) mouse plasma analyzed by Western blotting. The asterisk indicates a nonspecific band.

The stability of Z_HER2:2891_-Fc-MMAE in human and mouse sera was monitored by Western blotting with an anti-affibody antibody. The strong signal of the Z_HER2:2891_-Fc-MMAE conjugate was detected for each day of the 7-day incubation both in human and mouse plasma (Figure 6a,b). This indicates that in the above-mentioned conditions our construct remains relatively stable since the levels of the conjugate detected by the anti-affibody antibody were comparable between different days of the incubation.

## 3. Discussion

Antibodies are commonly used to generate new cytotoxic conjugates. These proteins are highly specific which ensures that the drug is delivered to target cells overexpressing tumor-associated antigens. However, due to the large size of these molecules (~150 kDa), their ability to penetrate solid tumors is limited [47]. Therefore, other macromolecules are also tested as drug carriers. The anti-HER2 affibodies labeled with radioisotopes have been widely investigated for molecular imaging of HER2-positive tumors [31,48,49,50]. These efforts have recently led to successful clinical trials of the second-generation anti-HER2 affibody loaded with ^111^In and ^68^Ga [51,52,53].

The anti-HER2 affibody armed with one MMAE molecule specifically targets HER2-positive cells and destroys them with a low nanomolar IC_50_ [19]. Here, we improved this conjugate by fusing the Fc fragment of IgG1 to Z_HER2:2891_ to expand its size and increase the number of the coupled drug molecules (Figure 1). The obtained Z_HER2:2891_-Fc construct run on SDS-PAGE gel under reducing conditions as a ~36 kD-protein (Figure 2b and Figure 3b) while the mass of the monomeric Z_HER2:2891_-Fc determined by MS was 33929.5 Da (Figure 2c). The band shift towards higher molecular weight observed on the SDS-PAGE gel and the ~1100 Da discrepancy between the apparent molecular mass of the Z_HER2:2891_-Fc monomer (33,929.5 Da) and its calculated molecular mass (32,833 Da) suggest that similar to other Fc fusions [41,54], the Fc fragment was glycosylated in CHO-S cells. Combining the anti-HER2 affibody with Fc allowed us to expand the molecular mass of the affibody from ~7 kDa to ~70 kDa, which is still about half of the antibody size. Such size should contribute to a slower kidney removal of our construct since the kidney filtration cutoff is around 40–60 kDa [33]. Additionally, the Fc domain prolongs the serum half-life of antibodies and Fc-fusion proteins due to pH-dependent binding to the neonatal Fc receptor (FcRn) which protects the proteins from endosomal degradation in the epithelial cells. The Fc-fusion bound to FcRn is then recycled back into circulation [34,36,38]. The ability of the Fc region to improve pharmacokinetics has been verified for numerous proteins including eleven Fc fusions approved by FDA till 2017 [55].

The Z_HER2:2891_-Fc construct specifically recognized HER2-positive cells, which was confirmed by microscopic images and a biochemical binding assay (Figure 3). The presence of Fc allowed us to conjugate MMAE molecules to four cysteines obtained by treating Z_HER2:2891_-Fc with a reducing agent to break interchain disulphide bonds prior to the conjugation process, as it has been described for many ADCs [56,57,58]. According to the analysis of mass spectra, one or two MMAE molecules were coupled to a single Z_HER2:2891_-Fc which resulted in up to four MMAE molecules per dimeric Z_HER2:2891_-Fc (Figure 4b). The Z_HER2:2891_-Fc-MMAE dimer underwent decomposition to monomer during the MS sample preparation and measurement (Figure 4b). However, the dimeric format of the conjugate was preserved during electrophoretic separation with a low amount of SDS in the running buffer (Figure 4c). This indicates that in physiological conditions Z_HER2:2891_-Fc-MMAE should remain dimeric.

We obtained the following IC_50_ values for cells treated with the Z_HER2:2891_-Fc-MMAE conjugate: 130 pM for SK-BR-3 (HER2+) cells, 1.87 nM for MDA-MB-453 (HER2+) cells, 45.67 nM for T-47-D (HER2-) cells and 98.22 nM for MDA-MB-231 (HER2-) cells (Figure 5 and Table 1). This translates into a significant increase in the sensitivity of HER2-positive cells towards Z_HER2:2891_-Fc-MMAE in comparison with their sensitivity to Z_HER2:2891_-DCS-MMAE loaded with a single auristatin E molecule (the 38- and 12-fold increase in the case of SK-BR-3 and MDA-MB-453 cells, respectively). Importantly, the sensitivity of the HER2 negative cells to Z_HER2:2891_-Fc-MMAE was elevated to a lesser extent (Figure 5 and Table 1). The higher efficacy of the Z_HER2:2891_-Fc-MMAE conjugate may also be caused by an enhanced internalization of this bivalent construct, which is in accordance with the results demonstrating that cross-linking or clustering of transmembrane receptors accelerates endocytosis [59].

Recently, we have generated an auristatin conjugate based on the bivalent Z_HER2:4_ affibody referred to as (Z_HER2:4_)_2_DCS-MMAE [21]. Z_HER2:4_ is an earlier version of the anti-HER2 affibody and binds HER2 receptor with the affinity three orders of magnitude lower than Z_HER2:2891_ [29,30]. The (Z_HER2:4_)_2_DCS-MMAE conjugate killed SK-BR-3 cells with a similar efficacy (IC_50_ = ~500 pM) as Z_HER2:2891_-Fc-MMAE. However, this bivalent construct is highly heterogeneous since it carries from zero to three MMAE molecules. Moreover, the size of (Z_HER2:4_)_2_DCS-MMAE is below 20 kDa which makes it prone to removal through kidney clearance. In this context, the Z_HER2:2891_-Fc fusion provides a better platform for a cytotoxic conjugate development than single and bivalent affibodies. Interestingly, the dimeric (Z_HER2:4_)_2_DCS affibody slightly affected the SK-BR-3 cell viability during a 4-day incubation [21]. This finding is in contrast to our preliminary observation that the Z_HER2:2891_ affibody fused to the Fc fragment enhances viability of the HER2 positive cells (data not shown). This effect of Z_HER2:2891_-Fc was not observed for MDA-MB-231 cells that do not overexpress HER2 receptor. We hypothesize that the Z_HER2:2891_-Fc dimer induces cell proliferation by activating HER2 signaling pathways. However, this possibility needs to be further investigated.

Ado-trastuzumab emtansine (Kadcyla, Genentech), which is one of the two ADCs currently approved for clinical use by the FDA, similarly to the Z_HER2:2891_-Fc-MMAE, targets HER2 receptor. In this ADC cytotoxic payload, DM1 (emtansine), is attached to lysine amines via the SMCC linker [60,61,62]. This conjugate carries 3.2–3.8 DM1 molecules per antibody on average [61,63] which is close to four that is the optimal drug to antibody ratio (DAR) proposed by Hamblett et al. (2004) [57]. However, the distribution of DM1 is not uniform since there are about 40 lysines that can be modified in an antibody affecting pharmacokinetics of the conjugate [64]. Our conjugate relied on MMAE coupling to the interchain cysteines, which results in defined conjugation sites. This strategy ensures batch-to-batch consistency in terms of MMAE location. Importantly, the efficacy of trastuzumab-DM1 tested on the SK-BR-3 cell lines is comparable to the cytotoxicity of (Z_HER2:4_)_2_DCS-MMAE [61].

The Fc part of IgG1 contributes to a cell killing-activity by three mechanisms: antibody-dependent cell-mediated cytotoxicity (ADCC), antibody-dependent phagocytosis (ADPh) and complement-dependent cytotoxicity (CDC) [65,66,67]. Notably, a recent study by Mobergslien et al. demonstrated that a fusion of a targeting peptide, referred as WN, with the Fc fragment activated innate immune cells (NK cells, monocytes, dendritic cells). Injection of this construct to 4T1 tumor-bearing BALB/c mice inhibited tumor growth and the tumor tissue was infiltrated by NK cells and T cells [54]. These findings suggest that the cytotoxicity of the proposed Z_HER2:2891_-Fc-MMAE conjugate can be further enhanced by its potential to trigger the immunological response directed against HER2-positive cancer cells. This possibility will be addressed in further studies.

## 4. Materials and Methods

### 4.1. Construction of the Z_HER2:2891_-Fc Expression Plasmid

The nucleotide sequence of Z_HER2:2891_ was amplified by PCR (Polimerase Chain Reaction) from the bacterial expression plasmid pDEST-Z_HER2:2891_-DCS [19] using the primers A: 5′-CTCTTCTTCCTGTCAGTAACGACTGGTGTCCACTCCGCCGAAGCCAAATATGCAAAA-3′ and B: 5′-TTGCGTCCGGATTTCGGTGCCTGGCTATCAT-3′. This PCR resulted in the DNA fragment encoding the last 12 amino acids of the signaling peptide, followed by the sequence encoding of the affibody and *Kpn2I* (*BspMII*) restriction site at the 3′ of the PCR product. The second round of PCR was carried out with primer C: 5′-CTCCAAGCTTTGAACCACCATGGAATGGAGCTGGGTCTT TCTCTTCTTCCTGTCAGTAACG-3′ and B and resulted in the DNA fragment comprising *HindIII* restriction site at the 5′, the sequence encoding all amino acids of the signal peptide, the affibody and *Kpn2I* (*BspMII*) restriction site at the 3’ of the PCR product. The PCR product and the pLEV113-ECD_FGFR2-Fc construct [41] were digested with HindIII and Kpn2I (BspMII) and subjected to ligation using T4 ligase. The resulting construct pLEV113-Z_HER2:2891_-Fc was verified by sequencing, propagated in DH10α *E. coli* cells and purified using Plasmid Maxi Kit (Qiagen, Hilden, Germany).

### 4.2. Protein Expression

The Z_HER2:2891_-Fc expression was carried out according to Sokolowska-Wedzina et al. (2014) with minor modifications [41]. Briefly, CHO-S cells (Invitrogen, Carlsbad, CA, USA) were cultured in PowerCHO-2CD medium (Lonza, Basel, Switzerland) supplemented with 8 mM *L*-glutamine and 1× penicillin/streptomycin solution (Biowest, Nuaillé, France). Cells were grown at 37 °C in a shaking incubator (140 rpm) with 8% CO_2_. The cells were diluted with fresh medium to density of 0.3 × 10^6^ cells/mL when the culture density reached 1–1.2 × 10^6^ cells/mL. One day before transfection the CHO-S cells were diluted to 0.6 × 10^6^ cells/mL. On the day of transfection culture was centrifuged and cell pellet were resuspended in ProCHO4 medium (Lonza, Basel, Switzerland) at 2 × 10^6^ cells/mL. Appropriate amount of plasmid (1.25 µg of DNA per 1 × 10^6^ cells/mL) was resuspended in 150 mM NaCl. The final volume of the DNA solution was 5% of the cell suspension volume. Appropriate amount of 1 mg/mL linear PEI (Polyscience, Warrington, PA, USA) was added to 150 mM NaCl aiming for 5 µg PEI per 1 × 10^6^ cells/mL. The final volume of the PEI solution was 5% of the cell suspension. The DNA and PEI solutions were mixed together, incubated at room temperature for 10 min and then added dropwise to the cell solution. The cells were incubated for 4 h in standard conditions (37 °C, 140 rpm, 8% CO_2_). After this time, the culture was diluted with an equal volume of PowerCHO-2CD supplemented with 8 mM l-glutamine and 2× penicillin-streptomycin solution (Biowest, Nuaillé, France) and incubated at 31 °C. On the second day following the transfection the culture was supplemented with an additional 4 mM l-glutamine and harvested on sixth day.

### 4.3. Protein Purification

The purification of Z_HER2:2891_-Fc was carried out according to Sokolowska-Wedzina et al. (2014) with minor modifications [41]. EDTA was added to the CHO-S cell culture to a final concentration of 2 mM and the culture was centrifuged (25 min at 15,000× *g*, 4 °C). Supernatant was collected, centrifuged (25 min at 15,000× *g*, 4 °C), filtered through 0.22-micron filter units and loaded onto a Protein A Sepharose column (GE Healthcare, Chicago, IL, USA) pre-equilibrated with PBS. The unbound fraction was removed and the resin was washed with buffer A: PBS, 200 mM NaCl, 0.1% Tween 20, 2 mM EDTA, pH 7.4 and then with buffer B: PBS, 500 mM NaCl, 2 mM EDTA, pH 7.4. Z_HER2:2891_-Fc was eluted with 100 mM triethylamine, and neutralized with 1 M Tris–HCl, pH 7.2. Fractions with the highest protein concentrations were collected, dialyzed against PBS, and concentrated using Centriprep 10 K centrifugal filter units (Mreck Millipore, Billerica, MA, USA). Protein purity was checked by SDS–PAGE and their identity was confirmed by mass spectrometry.

### 4.4. Mass Spectrometry

Z_HER2:2891_-Fc and its conjugate were detected by MS 4800 Plus MALDI TOF/TOF Analyzer (AB Sciex, Framingham, MA, USA) with sinapic acid (Sigma-Aldrich, Saint Louis, MO, USA) 10 mg/mL 50% acetonitrile 0.1% TFA as a matrix.

### 4.5. Western Blotting

Western blotting was performed as described in Sochaj-Gregorczyk et al. (2016) [19]. Primary antibodies used in this study include: the goat anti-affibody antibody (Affibody AB, Solna Sweden) (conjugate stability assay), the anti-Fc antibody-HRP (KPL, Milford, MA, USA) (expression and purification analysis, Z_HER2:2891_-Fc binding to SK-BR-3 and MDA-MB-231 cell surface), the mouse anti-gamma-tubulin monoclonal antibody (Sigma-Aldrich, Saint Louis, MO, USA) (Z_HER2:2891_-Fc binding to SK-BR-3 and MDA-MB-231 cell surface) and the mouse anti-HER2 (e2-4001) monoclonal antibody (Thermo Fisher Scientific, Waltham, MA, USA) (Z_HER2:2891_-Fc binding to SK-BR-3 and MDA-MB-231 cell surface). For detection of the goat and mouse primary antibodies, the secondary anti-goat antibody-HRP (Jackson ImmuoResearch, West Grove, PA, USA) and anti-mouse antibody-HRP (Jackson ImmuoResearch, West Grove, PA, USA) were used, respectively.

### 4.6. The Z_HER2:2891_-Fc-MMAE Conjugate Preparation

Z_HER2:2891_-Fc at the concentration of 0.5 mg/mL in PBS pH 7.2 was subjected to reduction with a 10 molar excess of tris-2-karboksyetylofosfine (TCEP) for 2 h at 37 °C. The reduced Z_HER2:2891_-Fc was incubated with a 16 molar excess of MC-Val-Cit-PABC-MMAE (maleimidocaprylvaline-citruline-*p*-amino-benzyloxycarbonyl-mono-methylauristatin E) (ChiroBlock, Wolfen, Germany) for 3 h with gentle mixing at 4 °C. The conjugation mixture was centrifuged at 20,000× *g* for 10 min. Unconjugated MMEA was removed using Zeba spin column (Thermo Fisher Scientific, Waltham, MA, USA). The efficiency of conjugation was monitored on 12.5% SDS-PAGE gel and by mass spectrometry. The conjugate was stored at 4 °C.

### 4.7. Z_HER2:2891_-Fc Labeling with FITC

50 µg fluorescein 5(6)-isothiocyanate (FITC) (Sigma-Aldrich, Saint Louis, MO, USA) was added to 500 µg of Z_HER2:2891_-Fc dissolved in PBS, pH 7.2. The sample was incubated in the dark for 3 h. In order to remove unconjugated FITC, the PD10 column (GE Healthcare, Chicago, IL, USA) was used.

### 4.8. Immunostaining

SK-BR-3 and MDA-MB-231 cells were allowed to grow in appropriate media on cover slips that were placed in a 6-well plates for two days. Cells were washed twice with PBS and then fixed with 4% paraformaldehyde for 10 minutes. After paraformaldehyde removal cells were washed 3 times with PBS and then incubated with PBS, 2% BSA, 0.01% Triton X-100 for 15 min at RT. Cells were washed twice with PBS, 0.01% Triton X-100 and incubated for 1 h with a mouse anti-HER2 (e2-4001) monoclonal antibody (Thermo Fisher Scientific, Waltham, MA, USA) or Z_HER2:2891_-Fc-FITC diluted in PBS, 0.01% Triton X-100. Following the incubation, cells were washed three times with PBS, 0.01% Triton X-100. Then a 1 h incubation with a secondary fluorescein (FITC) AffiniPure F (ab’) fragment donkey anti-mouse IgG (Jackson ImmunoResearch, West Grove, PA, USA) was carried out for the cells stained with the mouse anti-HER2 antibody. Following three washes with PBS, 0.01% Triton X-100 cover slips were mounted onto slides.

### 4.9. Microscopy

Microscopical analysis was performed as described in Sochaj-Gregorczyk et al. (2016) [19].

### 4.10. Cell Lines

The following breast cancer cell lines were used in this study: SK-BR-3, MDA-MB-453, MDA-MB-231 and T-47-D (American Type Culture Collection, Manassas, VA, USA). The MDA-MB-453, MDA-MB-231 and T-47-D cells were cultured in Dulbecco's Modified Eagle Medium, DMEM (Sigma-Aldrich, Saint Louis, MO, USA), and the SK-BR-3 in the McCoy’s 5A medium (Lonza, Basel, Switzerland). All media contained 2 mM l-glutamine and were supplemented with 10% FBS (Thermo Fisher Scientific, Waltham, MA, USA) and 1 x penicillin-streptomycin solution (Biowest, Nuaillé, France). The cells were cultured at 37 °C in 5% CO_2_ and humidified atmosphere. Upon reaching 90% confluence, the cells were passaged using Trypsin-EDTA solution (Thermo Fisher Scientific, Waltham, MA, USA)

### 4.11. Cytotoxicity Assays

The early-passage cells were seeded at the density of 5000 cells per well in a 96-well plate. The cells were allowed to attach and after 24 h different concentrations of the conjugate were added to the wells. After 96-incubation of continuous exposure to the Z_HER2:2891_-Fc-MMAE conjugate, the medium was replaced with a fresh medium containing 10% of AlamarBlue reagent (Thermo Fisher Scientific, Waltham, MA, USA). After 4 h, formation of a fluorescent reduced form of the dye was determined using EnVision Multimode Plate Reader (PerkinElmer, Waltham, MA, USA). The IC_50_ values were determined using the OriginPro8 software v8.0724 (OriginLab, Northampton, MA, USA).

### 4.12. Monitoring Stability of the Z_HER2:2891_-Fc-MMAE Conjugate in Mouse and Human Sera

For the stability assay, 10 µL of conjugate (0.5–1 mg/mL solution) was added to 90 µL of mouse (Invitrogen, Carlsbad, CA, USA) or human serum (Sigma-Aldrich, Saint Louis, MO, USA) and incubated for 6 days at 37 °C. The eppendorf tubes were sealed with parafilm to minimize evaporation. The 10 µL samples were taken every 24 h, mixed with reducing Laemmli sample buffer, boiled (96 °C, 10 min) and separated on 12% SDS-PAGE gel. Upon electrotransfer the membrane was stained with Ponceus S solution and subjected to Western blotting with the goat anti-affibody antibody (Affibody AB, Solna, Sweden) and secondary anti-goat antibody-HRP (Jackson ImmuoResearch, West Grove, PA, USA).

## Figures and Tables

**Table 1:** The cytotoxicity of Z_HER2:2891_-Fc-MMAE in comparison to the previously published IC_50_ values obtained for the Z_HER2:2891_-MMAE conjugate. IC_50_ results are expressed as mean ± SEM.

	Cytotoxic Agent	SK-BR-3 (10) *	MDA-MB-453 (8) *	T-47-D (5) *	MDA-MB-231 (2) *
IC_50_ (nM)	free MMAE [19]	1.76 ± 0.26 (*n* = 3)	1.27 ± 0.46 (*n* = 3)	3.25 ± 1.06 (*n* =3)	5.21 ± 1.46 (*n* = 3)
	Z_HER2:2891_-MMAE [19]	5.16 ± 1.09 (*n* = 4)	24.83 ± 5.62 (*n* =3)	135.55 ± 22.7 (*n* = 4)	161.53 ± 49.9 (*n* = 3)
	Z_HER2:2891_-Fc-MMAE	0.13 ± 0.01 (*n* = 7)	1.87 ± 0.27 (*n* = 4)	45.67 ± 7.43 (*n* = 3)	98.22 ± 30.9 (*n* = 7)

* The erbB2 gene expression values according to HerceptinR, a database for Herceptin resistance [46].
